# MRI assessment of intrinsic neural timescales in male alcohol use disorder patients

**DOI:** 10.3389/fpsyt.2026.1736813

**Published:** 2026-02-19

**Authors:** Weijian Wang, Xinyu Wang, Yimeng Kang, Wenjing Li, Yichen Guo, Hui Zhang, Jiawen Tian, Longyao Ma, Bohui Mei, Mengzhe Zhang, Yarui Wei, Yong Zhang

**Affiliations:** 1 Department of Magnetic Resonance Imaging, The First Affiliated Hospital of Zhengzhou University, Zhengzhou, China; 2 Department of Radiology, Union Hospital, Tongji Medical College, Huazhong University of Science and Technology, Wuhan, China

**Keywords:** alcohol use disorder, default networks, intrinsic neural timescale, neurodynamic changes, visual attention network

## Abstract

**Background:**

Alcohol use disorder (AUD) is a chronic, relapsing condition marked by compulsive drinking, imposing a significant burden on both the individual and their environment. The intrinsic neural timescale (INT) is determined through the assessment of autocorrelation of the brain activity in resting-state functional magnetic resonance imaging, serving to elucidate the diversity of neural timescales.

**Method:**

This study involved 55 alcohol-dependent patients and 33 non-drinking healthy controls (HCs) matched for age, sex, and hand-use habits. We calculated the INT by evaluating the strength of autocorrelation in resting-state brain activity, and subsequently compared between-group differences in INT. We also analyzed the correlation between abnormal INT and clinical characteristics.

**Result:**

The AUD group exhibited significantly longer INT in the left and right thalamus when compared to the HC group. Additionally, significantly shorter INT was observed in the calcarine cortex in the AUD group in comparison to the HC group.

**Conclusion:**

Our study identified abnormalities of the brain activity at the resting state in alcohol-dependent patients by means of an INT approach, which may provide more insight into the neural mechanisms of alcohol dependence.

## 1 Introduction

Alcohol use disorder (AUD) is a chronic relapsing condition marked by compulsive drinking, imposing a significant burden on both the affected individual and their immediate environment (1). Recently, it is estimated that more than 10% of the population was affected (2). Recognized as one of the primary causes of global morbidity and mortality, AUD poses a substantial public health challenge (3). Despite the availability of evidence-based psychotherapeutic and pharmacologic interventions, a considerable portion of individuals with AUD fail to sustain long-term improvement. Detoxified AUD patients have reported relapse rates of up to 85% without continued treatment (4, 5). Further still, even following extended hospitalization, between 20-80% of AUD patients resume drinking within 12 months of discharge from the hospital (6). Imaging biomarkers for AUD are increasingly important for monitoring progress in clinical trials (7).

Many previous multimodal medical imaging studies have revealed several satisfying findings about AUD, including structural and functional alterations, static and dynamic, which have been very useful in revealing neurobiological markers of AUD (8, 9). Böhmer et al. used a graph theory approach and revealed that at the whole-brain level, relapsers processed information in segregated ways within functionally interconnected regions, rather than efficiently distributing information throughout the brain; at the local level, relapsers exhibit dysfunctional integration and decoupling between multiple brain systems, particularly involving cortical and subcortical brain regions associated with higher executive functions, sensory and reward processing (10). AUD has previously been linked to gray matter differences in the prefrontal cortex and reward-processing areas (11, 12). Thus, studies of changes in activity in brain regions may provide a further wealth of information.

The importance of the destruction of localized areas of the brain is not something we can ignore. As recently reported, combining the amplitude of low frequency fluctuation (ALFF) or regional homogeneity (ReHo) with “sliding-window” approaches, the dynamic ALFF/ReHo method provides a new avenue to depict time-varying local brain activity (13) and has been used in patients with depression (14), generalized anxiety disorder (15), cigarette smoking (16, 17), schizophrenia (18), conduct disorder (19, 20), epilepsy (21) and so on. We may speculate that analyzing time-varying patterns of local brain activity holds promise for understanding the neuropathological mechanisms of psychiatric disorders. Although studies have demonstrated the presence of abnormal spontaneous brain activity in patients with AUD (22), it remains uncertain whether precise intrinsic neural timescales (INT) within local brain regions manifest abnormalities in individuals with AUD.

To deepen our understanding of local intrinsic brain activity, Watanabe et al. (23) proposed a new method called INT, which calculates the neural heterogeneity by evaluating the degree of autocorrelation of the resting state brain activity, and is used to characterize the length of time that neurological information may be stored in the neural regions, which is considered to characterize the functional hierarchy of the local brain region (24–30). INT is thought to represent a temporal window for the integration of neural information, reflecting an ability to accumulate information over long periods of time. Through autocorrelation function analysis, it quantifies the persistence and integration window of neural activity time series, directly characterizing the nervous system’s temporal integration capacity for information. This provides a direct quantitative metric for understanding the temporal mechanisms of brain information processing (31). In contrast, traditional rs-fMRI studies (such as ALFF, ReHo, and functional connectivity) primarily describe the spatial characteristics or signal amplitude of neural activity (32, 33). Furthermore, while conventional metrics fail to reveal cross-brain temporal specialization patterns, INT presents a hierarchical temporal gradient distribution across the entire brain (34). For instance, INT values progressively increase from primary sensory cortices to higher-order association cortices, capturing the “cross-cortical temporal gradient” that traditional metrics cannot quantify. This provides crucial evidence for functional hierarchy abnormalities throughout the brain. We hypothesize that patients with AUD exhibit abnormalities in their INT. This phenomenon may directly reveal disruptions in the functional hierarchy of relevant brain regions, providing direct evidence for systematically investigating the neurobiological mechanisms underlying AUD.

Longer INT represent higher levels of neural hierarchies, such as the prefrontal and parietal lobes, which require longer timescales than peripheral sensory areas to enable these higher-order cerebral cortices to integrate a variety of information (24, 28), relying on stable cognitive processes that accumulate information over time (25). In contrast, shorter INT indicate more random brain activity, which allows for rapid responses to changing stimuli in the environment (24). This new pathway has been successfully applied to study the dynamics of localized brain regions in the resting state of patients with autism spectrum disorders (23) and schizophrenia (35). In particular, Watanabe et al. concluded that faster INT in the sensorimotor cortex correlate significantly and reproducibly with atypical behaviors in autism. However, to date, no local intrinsic neurodynamic have been studied to determine whether abnormal INT was exhibited in patients with AUD.

Thus, this study aimed to: (1) assess whether abnormal INT is present in the brain of AUD patients; (2) explore the correlation between abnormal INT in AUD patients and clinical symptoms.

## 2 Materials and methods

### 2.1 Participants

This study randomly recruited 55 AUD patients and 32 HCs (**Table 1**). Individuals with AUD were recruited from inpatient wards, internet postings, and advertisements. Meanwhile, participants in the HC group were volunteers from the local communities. Because body mass index (BMI) and smoking are highly associated with alcohol (36, 37), we also recorded the BMI data of all subjects and recorded whether the participants were smokers (in part of subjects). We also recorded the duration of alcohol drinking, alcohol by volume, the frequency of alcohol drinking per week, the amount of alcohol drinking, mean amount of pure alcohol per week (amount of alcohol drinking per day × frequency of alcohol drinking per week × alcohol by volume × 0.8), and the willingness of abstinence (0 for no willingness of abstinence and 1 for the willingness of abstinence). All subjects were men in this study. Inclusion criteria of AUD patients were: (1) meeting the DSM-V criteria for AUD based on the clinical assessment of the principal investigator; (2) ranging in age from 18 to 65 years old; (3) drinking on average more than 14 units of alcohol per week according to the U.K. Chief Medical Officers (38); (4) no medical history of neurological system or medical diseases; (5) could understand and consent to study procedures. Exclusion criteria for both AUD and HCs were: (1) having a history of psychiatric, neurological, or physical disorder; (2) having contraindications for magnetic resonance imaging (MRI); (3) reporting currently taking centrally active medications. The severity of AUD was assessed using the alcohol dependence scale (ADS), alcohol use disorder identification test (AUDIT), cut down, annoyed, guilty, eye-opener (CAGE), alcohol, smoking, and substance use involvement screening test (ASSIST), obsessive-compulsive drinking scale (OCDS), clinical institute withdrawal assessment for alcohol dependence, revised (CIWA-Ar), and Michigan alcoholism screening test (MAST). We measured cognitive impairment of AUD patients using the Montreal cognitive assessment (MoCA).

**Table 1:** The demographic and clinical data of AUD and HC.

Demographics	AUD (n = 55)	HC (n = 32)	*P* values
Age (years, SD)	45.56 (8.88)	42.47 (12.34)	0.090
BMI (kg/m^2^, SD)	25.37 (2.92)	26.31 (2.21)	0.076
Sex			
Female	0	0	–
Male	55	32	
Smoking status (AUD: n = 49; HC = 17)			
Smoker	32	17	–
Non-smoker	17	–	
Duration of drinks (years, n = 29, SD)	20.14 (9.89)	–	–
Alcohol by volume (%, n = 28, SD)	51.71 (1.08)	–	–
Frequency (days/week, n = 30, SD)	4.09 (1.91)	–	–
Amount of drinks (ml/day, n = 30, SD)	238.33 (108.82)	–	–
Mean amount of alcohol (g/week, n = 30, SD)	522.38 (333.83)	–	–
CAGE (n = 33, SD)	1.18 (0.98)	–	–
AUDIT (n = 34, SD)	19.97 (7.22)	–	–
CIWA-Ar (n = 41, SD)	8.37 (7.02)	–	–
ASSIST (n = 30, SD)	21.20 (7.22)	–	–
MAST (n = 31, SD)	15.00 (8.58)	–	–
ADS (n = 41, SD)	12.76 (6.66)	–	–
OCDS (n = 31, SD)	14.42 (9.95)	–	–
MoCA (n = 29, SD)	28.24 (1.27)	–	–

ADS, alcohol dependence scale; AUD, alcohol use disorder; AUDIT, alcohol use disorder identification test; ASSIST, alcohol, smoking, and substance use involvement screening test; BMI, body mass index; CAGE, cutdown, annoyed, guilty, eye-opener; CIWA-Ar, clinical institute withdrawal assessment for alcohol dependence, revised; HC, healthy control; MAST, Michigan alcoholism screening test; MoCA, Montreal cognitive assessment; OCDS, obsessive compulsive drinking scale.

### 2.2 Data acquisition

All subjects were scanned using a 3.0T MRI scanner (MAGNETOM Prisma, SIEMENS, Germany) with a 64-channel receiver array head coil. Head motion and scanner noise were reduced using foam padding and earplugs. All participants were asked to remain alert with their eyes closed. We collected resting-state functional MRI (rs-fMRI) data from all participants. Functional images were acquired transversely with gradient spin echo planar imaging (EPI) sequence with the following settings: TR/TE = 1000/30 ms, slice number = 52, slice thickness = 2.2 mm, slice gap = 0.4 mm, flip angle = 70°, field of view = 17.6 × 17.6 cm^2^, number of averages = 1, matrix size = 64 × 64, voxel size = 2.75 × 2.75 × 2.2 mm^3^. A total of 400 volumes were collected, resulting in a total scan time of 6.67 mins.

### 2.3 Data preprocessing

The functional images were preprocessed using Data Processing Assistant for Resting-State fMRI (DPARSF) programs (39), which are based on Statistical Parametric Mapping 12 (SPM12, http://www.fil.ion.ucl.ac.uk/spm) and MATLAB 2018b (Math Works, Natick, MAUSA). The first 10 volumes were discarded due to unsteady magnetization. Slice-timing and realignment were performed. We excluded the subjects whose head motion with translational or rotational motion was higher than 3 mm or 3°. Then, data were spatially normalized to the Montreal Neurological Institute template (resampling voxel size = 3 × 3 × 3 mm^3^), detrended, and filtered (0.01–0.08 Hz). Image volumes with frame wise displacement (FD) >0.5 mm, as well as 1 back and 2 forward neighbors were scrubbed to further reduce the effect of head motion and were replaced by using the method of spline interpolation. Nuisance covariates were regressed (including Frist on 24 head motion parameters and white matter and cerebrospinal fluid signals), and based on previous studies and experience, whole-brain signals are not recommended to regress (40); Therefore, we did not perform regression on the global signal.

### 2.4 Intrinsic neural timescale map

Building upon a previous study (23, 41), the preprocessed fMRI data were utilized to assess the INT for each voxel across the whole brain of each participant as follows. First, an autocorrelation function (ACF) of the rs-fMRI signal of each voxel (time bin = TR) was estimated, and then the sum of ACF values in the initial period where the ACF values were positive was calculated. The upper limit of this period was set at the point where the ACF reached zero for the first time. After repeating this procedure for every voxel, the brain map was spatially smoothed (FWHM = 6 mm) to improve the signal-to-noise ratio, and a Z-transformed brain map was obtained to eliminate individual differences within groups. This whole-brain map was used as an INT map in which the value at each voxel was equal to the INT of the brain region.

### 2.5 Correlations between intrinsic neural timescale and symptom severity

The mean INT values of all voxels within each cluster from the corrected statistical map were calculated, and their associations with clinical measures (ADS, AUDIT, CAGE, OCDS, CIWA-Ar, ASSIST, MAST and MoCA) were assessed.

### 2.6 Statistical analysis

A Shapiro–Wilk test was used to check the normality of demographic, clinical, and head motion data for each group. The Wilcoxon signed-rank test was used to assess differences of nonnormally distributed data between groups and the *X^2^* test was used to compare categorical data between groups with IBM SPSS statistics19.0. Two-sample *t* tests were also performed to compare the group differences in INT between the AUD and HC groups, with age, sex and mean FD as covariates. The statistically significant threshold was set at voxel-wise *P* < 0.005, cluster-wise *P* < 0.05, and the minimum cluster size of 94 voxels after Gaussian random field (GRF) correction. Correlations between intrinsic timescales and clinical scales were assessed using Spearman’s rank correlation coefficients for measures that were significantly associations.

## 3 Results

### 3.1 Demographic, clinical and head motion data

For the AUD and HC groups, no significant between-group differences in age (*Z* = -1.696, *P* = 0.09), BMI (*Z* = -1.778, *P* = 0.076) or sex were found (Details in **Table 1**). And we did not detect any significant differences between the groups in terms of mean FD (Z = −1.743, P = 0.081) or in the number of image volumes scrubbed (Z = −1.166, P = 0.247).

### 3.2 Abnormal intrinsic neural timescales between the AUD and HC groups

**Figure 1:**
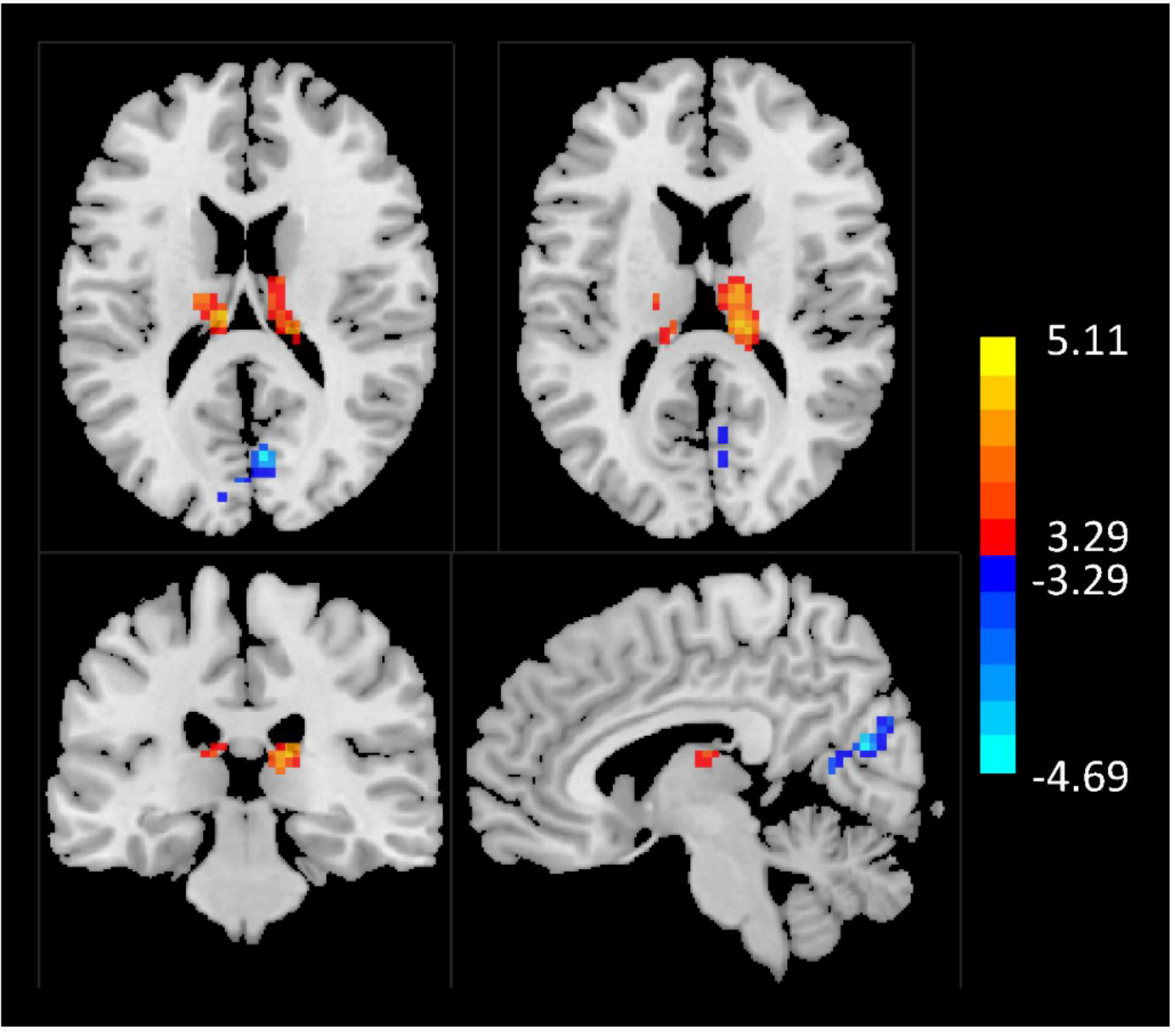
AUD showed significant differences in INT compared to healthy subjects. Areas of increased INT are shown in red. Areas of decreased INT are shown in blue. AUD, alcohol use disorder; INT, intrinsic neural timescale.

The AUD group had significantly longer timescales in the left and right thalamus compared with the HC group (**Table 2**; **Figure 1**). A significantly shorter timescale in the calcarine cortex was also found in the AUD group compared to the HC group (**Table 2**; **Figure 1**).

**Table 2:** Differences between the AUD and HC groups in the intrinsic neural timescale.

Between-group differences	Cluster	Regions	Hemisphere	Cluster size (voxels)	Peak MNI coordinate			Peak *F* values
					X	Y	Z	
AUD > HC	1	Thalamus	L	52	-9	-21	18	5.10
	2	Thalamus	R	87	15	-27	15	5.11
AUD < HC	3	Calcarine cortex	R	56	33	60	21	4.34

AUD, alcohol use disorder; L, left; HC, healthy control; MNI, Montreal Neurological Institute; R, right.

### 3.3 Correlations between intrinsic neural timescale and symptom severity in the AUD group

**Figure 2:**
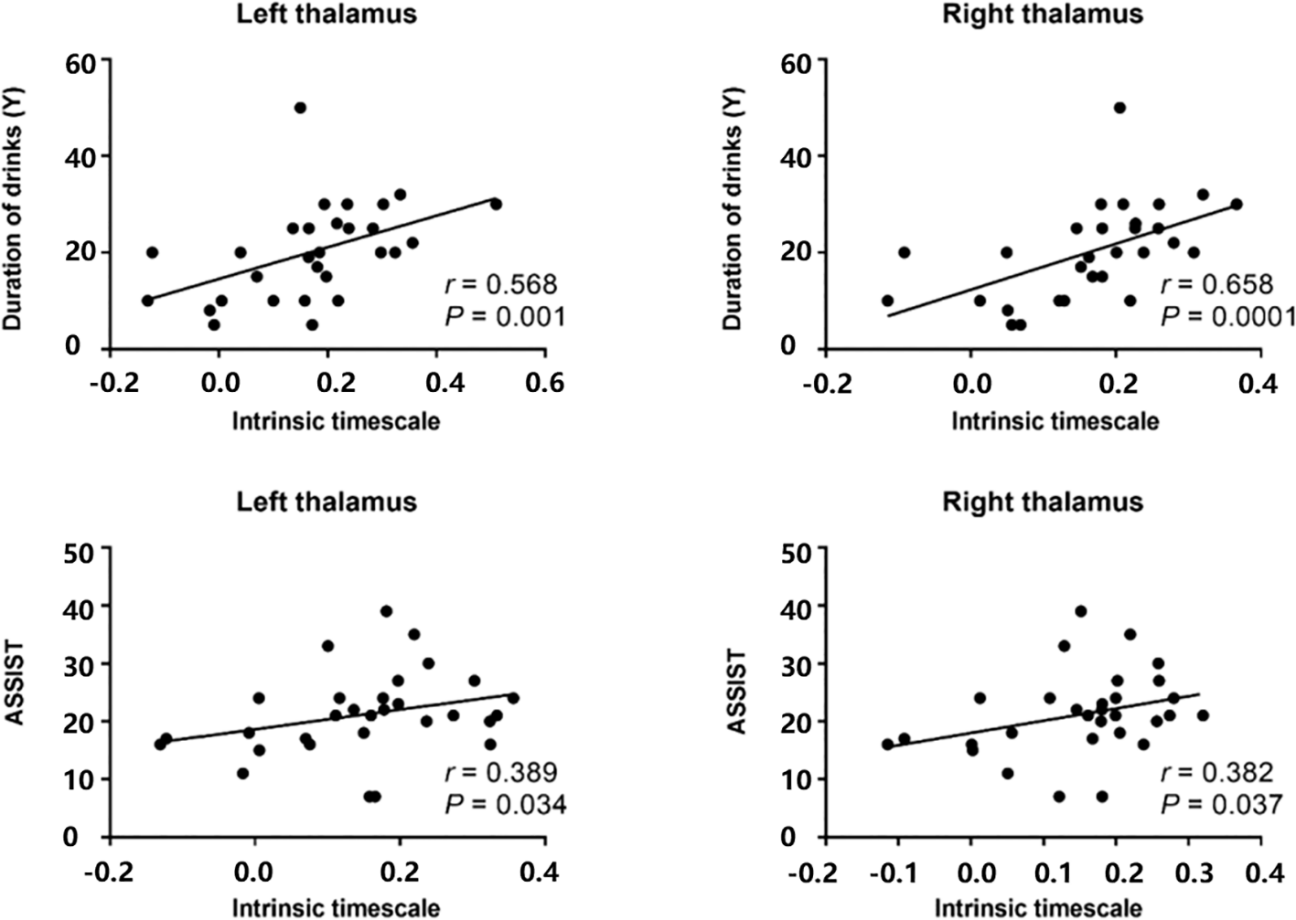
Correlations between intrinsic timescales and clinical measures in alcohol-dependent patients. The intrinsic time scale in the left thalamus was positively correlated (r=0.568, p=0.001) with duration of drink, and the intrinsic timescale in the right thalamus was also positively correlated (r=0.658, p=0.0001) with duration of drink. The intrinsic timescale in the left thalamus was positively correlated (r=0.389, p=0.034) with ASSIST scores, and the intrinsic timescale in the right thalamus was also positively correlated (r=0.382, p=0.037) with ASSIST scores. AUD, alcohol use disorder; ASSIST, alcohol, smoking, and substance use involvement screening test.

After correlating the mean intrinsic timescale values derived for all voxels within each cluster with clinical measures, we found that correlations between intrinsic timescales and clinical measures in alcohol-dependent patients The INT of the left thalamus was positively correlated with drinking duration (*r* = 0.568, *P* = 0.001), and the INT of the right thalamus was positively correlated with drinking duration (*r* = 0.658, *P* = 0.0001). The INT of the left thalamus was positively correlated with ASSIST score (*r* = 0.389, *P* = 0.034), as was the INT of the right thalamus (*r* = 0.382, *P* = 0.037) (**Figure 2**).

## 4 Discussion

In this study, we delved into the INT, which are linked to the functional hierarchy of the brain in individuals with AUD. Through an analysis of the time-dependent magnitude of the autocorrelation function in rs-fMRI data, our research uncovered abnormal INT in the bilateral thalami and the calcarine cortex of male AUD patients. Prior studies have also emphasized the crucial role of the thalamus in addiction research, as it serves as the nexus of numerous brain circuits involved in cognitive functions, emotional processing, and behavioral regulation (42). Furthermore, our findings indicated a significant correlation between the INT values of bilateral thalami and the duration of alcohol consumption and ASSIST scores. Collectively, these discoveries offer fresh insights into the neuropathological mechanisms of AUD.

In addiction research, it has been demonstrated that exposure to addictive substances leads to an increase in dopamine levels within reward circuits, which encompass the striatum (such as the caudate and pallidum) as well as the lateral and medial PFC (43). Dopamine connections in the midbrain run from the pallidum to the thalamus and then from the thalamus to the frontal lobes, so that in the thalamus not only existent inputs are included but also output projections (44). The thalamus plays a central role in perceptual integration, cognition, emotional processing (45), and executive functioning (46), acting as a relay station between the striatum and the cortex (47). Individuals facing challenges with emotional regulation often resort to inappropriate behaviors to avoid or alleviate negative emotions, potentially leading to the development of psychopathology (48, 49). Thus, individuals with AUD may turn to alcohol consumption when experiencing negative emotions as a means of adapting to adversity and seeking relief from emotional distress (50, 51). This is also consistent with previous research. Simultaneously, the thalamus monitors prefrontal activity through thalamocortical inputs, and then integrates this information with signals from the automaton and sensorimotor systems (52). Alterations in thalamocortical circuitry may consequently disrupt the integration of motivational and sensorimotor information in individuals with AUD, potentially impairing their goal-directed systems and leading to heightened reliance on habitual systems. Past studies have described changes in brain signaling over time in AUD subjects through rs-fMRI studies, examining not only local functional connectivity but also long-range connectivity. ReHo analysis is commonly used to estimate local functional connectivity, while resting state functional connectivity (rs-FC) analysis is used to estimate long-range connectivity (53). The studies found that abnormalities in local and remote connectivity were found in the frontal gyrus, cingulate cortex, caudate nucleus, thalamus, temporal gyrus, occipital gyrus, and cerebellum-cerebellum (54–57). The INT in a particular brain region of an AUD patient, whether shorter or longer, corresponds to a shorter or longer duration for the patient to store neural information in that region. This may help to explain why patients with AUD are more prompt and persistent in their engagement with alcohol-related stimuli (24, 25, 28), as well as why they might experience exacerbated deficits in inhibiting responses to such stimuli (58). Abnormal signals in these regions may indicate that there is random activity in the brain, causing weaker autocorrelation, which may be responsible for the phenomenon described above.

In addition, excessive alcohol consumption has been shown to affect various aspects of visual processing such as visual short-term memory, depth perception, contrast sensitivity, visual distractor attention, spatial information processing, and visual acuity to moving stimuli (59). The calcarine cortex plays an important role in visual information integration and attentional processing, and the calcarine cortex is part of the visual attention network (60, 61). Previous studies have also demonstrated the presence of extensive structural and functional alterations in the visual network in subjects with AUD, resulting in a decrease in visual function pairs (9, 59, 62). As with the above, our findings suggest that AUD subjects exhibit lower INT in the calcarine cortex, which is involved in the visual network, which may be explained by the fact that AUD can have deleterious effects on visual function. Previous reports have shown the existence of top-down attentional bias mechanisms in visual cortex (63). Based on the hypothesis that the dopamine system also has the capacity to draw attention to salient stimuli (64), substance-addicted individuals may exhibit changes in attentional processing when confronted with alcohol-related or drug-related cues. Extensive research suggests that substance-dependent individuals who allocate attention to substance-related cues at the expense of other environmental information and/or in the presence of alcohol- or drug-related cues will experience disruptions in their ability to focus attention on other stimuli (65). Attentional bias to alcohol-related cues can be observed in alcohol-dependent patients and social drinkers (66). In our study, reduced intrinsic timescales in the calcarine cortex implied lower levels of neural hierarchy, more randomized brain activity, and more pronounced stimulation of environmental changes, potentially suggesting a decline in visual skills in the visual network of alcohol-dependent patients.

Of note, our results found that alterations in the INT of the bilateral thalamus may be related to the clinical outcome of patients with AUD, as both INT values of the bilateral thalamus were positively correlated with the ASSIST score. This means that the higher the INT values, the higher the ASSIST scores of the patients, indicating a higher degree of risk of alcohol for AUD subjects. Overall, ASSIST scores may be the explanation for achieving and maintaining abstinence in AUD subjects, contributing to future research on AUD.

The functional hierarchy of the brain involves multiple systems in the brain, and abnormal intrinsic timescales of multiple systems in the AUD may also refer to abnormal functional hierarchies of the brain. Abnormal intrinsic timescales have also been found in several neurological or psychiatric disorders, such as in studies of schizophrenia (67), autism (23), epilepsy (68), and depression (69), all of which suggest that intrinsic timescales may bring new explanations for abnormal localized neurodynamics in the brains of patients with certain disorders. We therefore suggest that intrinsic timescales may also be indicators that can be considered for exploring neural substrates.

Additionally, INT abnormalities may provide valuable neuroimaging targets for clinical assessment and treatment monitoring of AUD. This study demonstrates that bilateral thalamic INT prolongation positively correlates with drinking duration and ASSIST scores. If this finding proves consistent, it could partially address the subjective limitations of existing scales, potentially establishing it as an objective biomarker for assessing AUD severity. And as a central hub of reward circuits and cognitive control networks, thalamic INT abnormalities may reflect patients’ attentional bias toward alcohol-related cues and deficits in impulse control. This characteristic could provide potential targets for developing personalized follow-up and intervention strategies for patients post-treatment.

However, current research is limited to male participants, and our findings should not be directly generalized to female patients with AUD. A growing body of evidence highlights gender-specific neurobiological differences in alcohol dependence: for example, a study by the ENIGMA Addiction Working Group (12) demonstrated that male and female individuals with alcohol use disorder exhibit distinct gray matter alterations in the hippocampus and amygdala—key regions involved in reward processing and emotional regulation. Sex differences in dopamine system function may also contribute to divergent neural responses to alcohol exposure (43). Additionally, hormonal fluctuations in women, such as estrogen-related dopaminergic neuromodulation in alcohol responses (70), may further alter INT and its association with the severity of alcohol use disorder. These sex-specific neurobiological characteristics suggest that the abnormal intrinsic neural timescale patterns observed in the bilateral thalamus and cingulate cortex may not fully reflect the neural mechanisms underlying alcohol use disorder in women. Future studies should incorporate female cohorts to validate and extend our findings.

### 4.1 Limitations

This study does have some limitations. Firstly, the small sample size may potentially reduce the persuasiveness of the findings. Additionally, the study’s subjects consisted solely of male Chinese patients, so this study doesn’t cover the entire spectrum of individuals with alcohol consumption habits. Future research should therefore employ gender-balanced designs to investigate whether abnormalities in the INT during AUD exhibit gender-specific patterns. Finally, the current study is cross-sectional in nature. Moving forward, it is essential to incorporate longitudinal data to complement and further understand the alterations in the INT of the brain in individuals with alcohol consumption habits.

### 4.2 Conclusion

In conclusion, our study identifies abnormalities in brain activity in AUD patients through an intrinsic time-scale approach. We found a higher INT in bilateral thalami and a shorter INT l in the calcarine cortex in male AUD patients compared to non-drinkers, which may indicate decreased functioning of male AUD patients in the involvement of the reward network and the visual attention network, which may play an important role in the understanding of failure to abstain from alcohol in AUD subjects. In summary, exploring the changes in neural activity of male AUD patients in the resting state by means of this index may provide a deeper understanding of the neural mechanisms of alcohol dependence.

## Data Availability

The original contributions presented in the study are included in the article/supplementary files, further inquiries can be directed to the corresponding author/s.
